# Supplementary material for: Insecticidal Efficacy of Microbial-Mediated Synthesized Copper Nano-Pesticide against Insect Pests and Non-Target Organisms
Source: Int J Environ Res Public Health. 2021 Oct 8;18(19):10536. doi: 10.3390/ijerph181910536 (PMC8508597; doi:10.3390/ijerph181910536)
Supplement: Supplementary file 1 [file ijerph-18-10536-s001.zip › ijerph-1292487-supplementary.pdf]

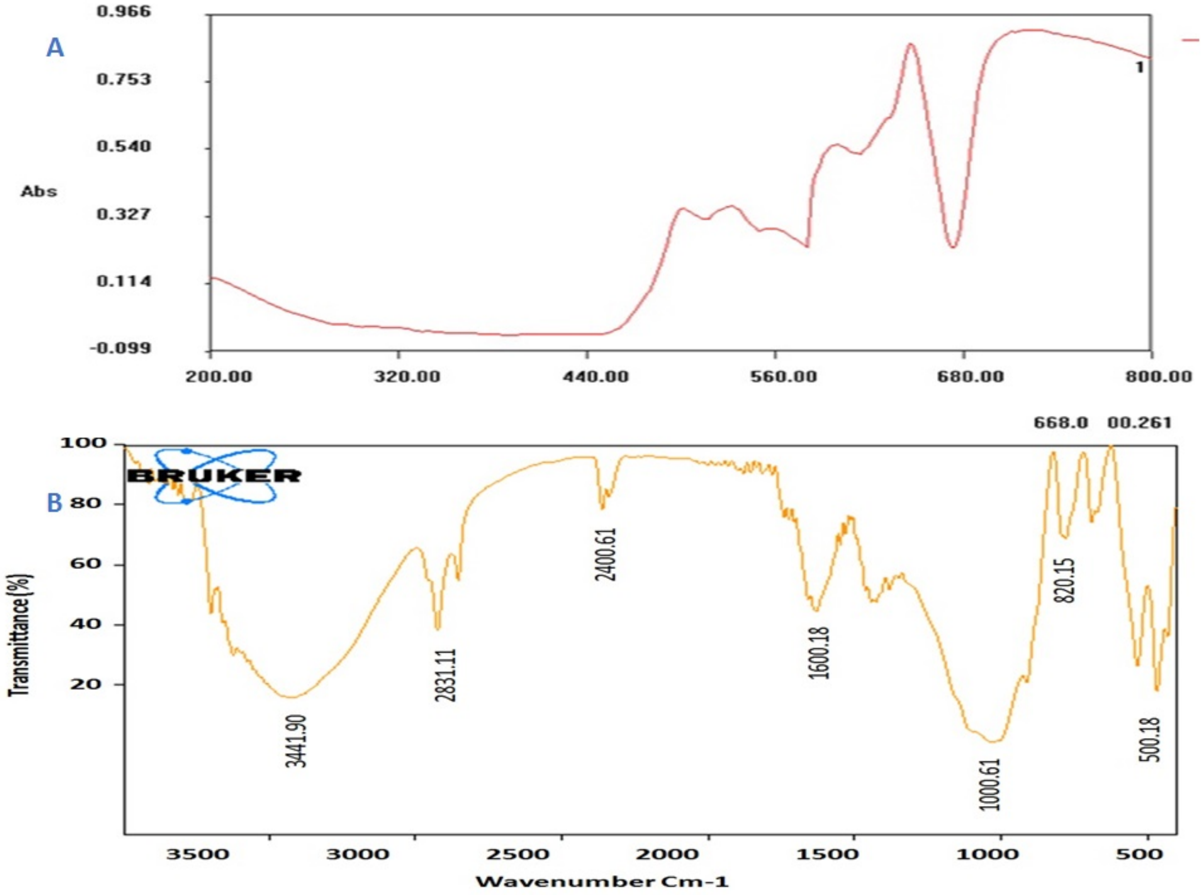

Figure S1: (**A**) UV–visible Spectrophotometer absorption range at 670 nm; (**B**) FT-IR analysis shows a several functional groups from green cluster copper nano-pesticide synthesized from *M. robertsii*

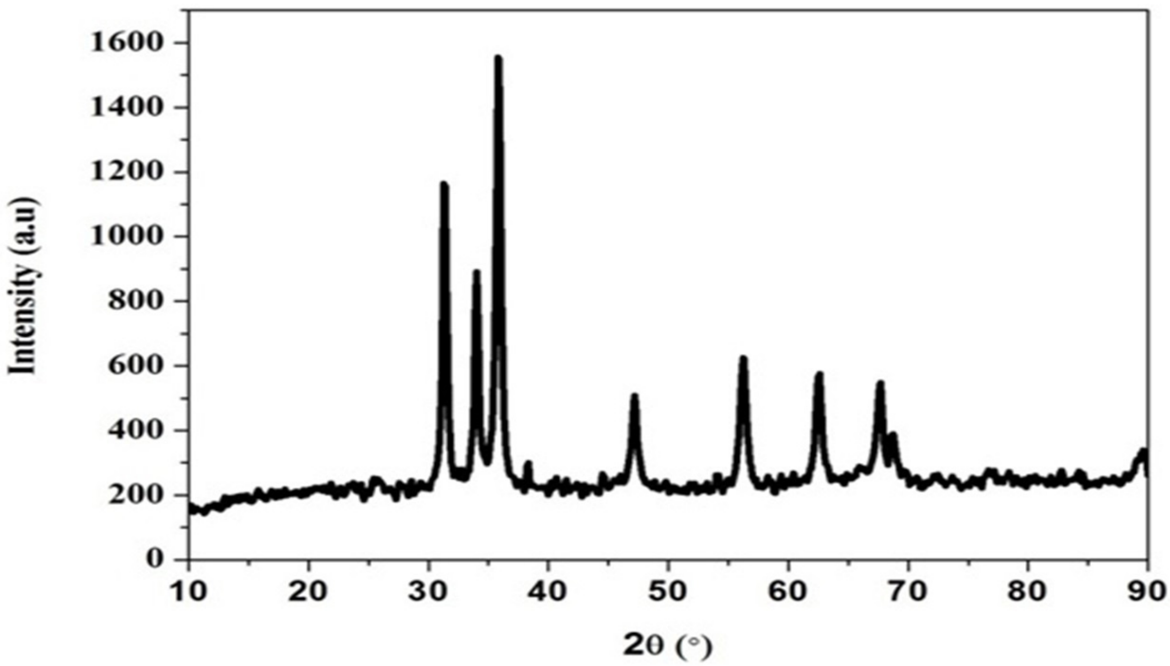

Figure S2: XRD shows 3 strongest peak values at 29.4389, 35.1523 and 36.8562 this strongest peak value tells about the crystalline structure of copper nano-particles

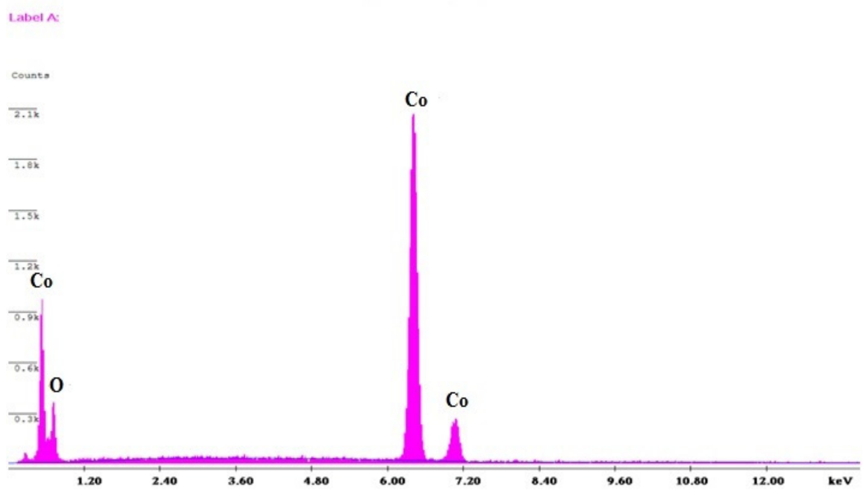

Figure S3. EDaX image gave an exact elementary composition and the ranges from 0 to 20 keV. It shows some elements such as O and CU.

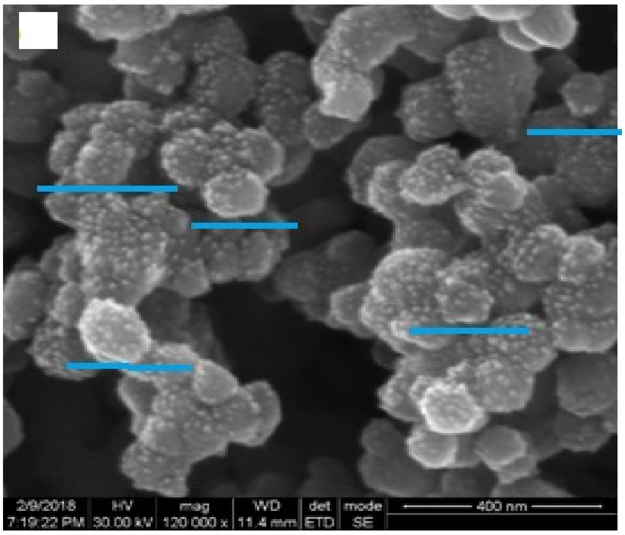

Figure S4: SEM analysis of green synthesis CuNPs that show sizes ranging from 15.67 nm–62.56nm.

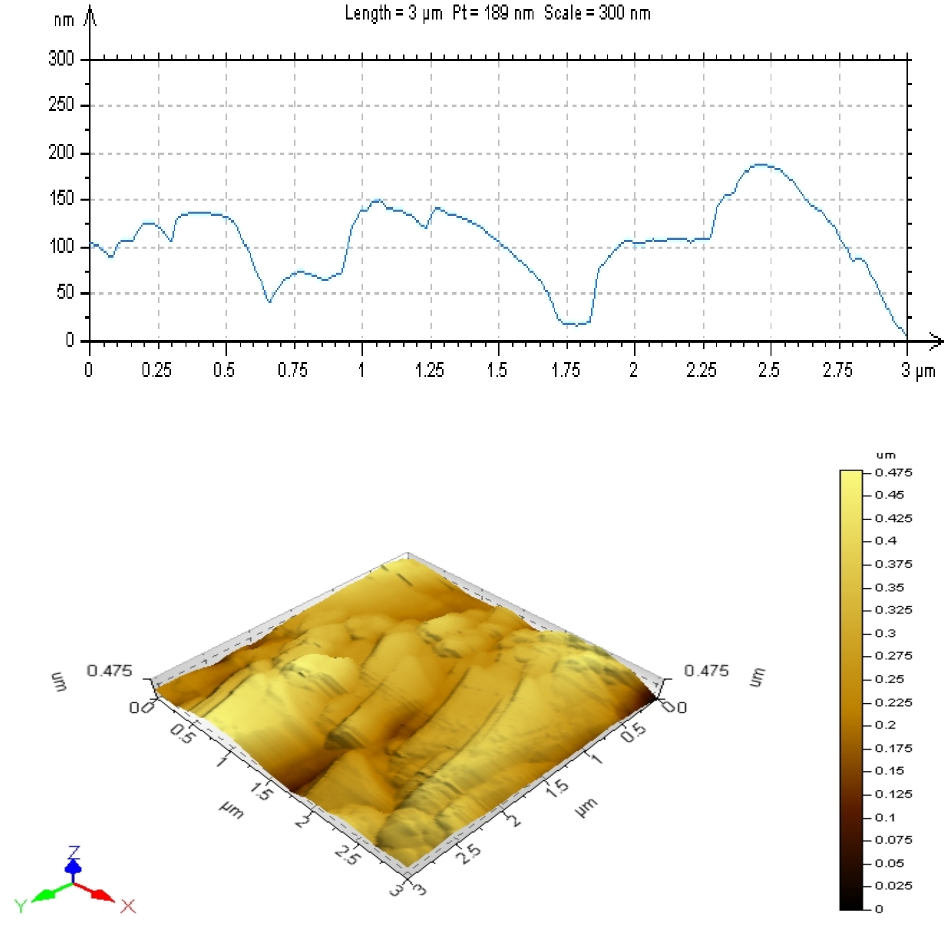

Figure S5. AFM analysis of green cluster copper nano-pesticide synthesized *M. robertsii*, A. 2.5µm resolution studies of 0–0.475 nm size, spherical shaped, and poly dispersed particles

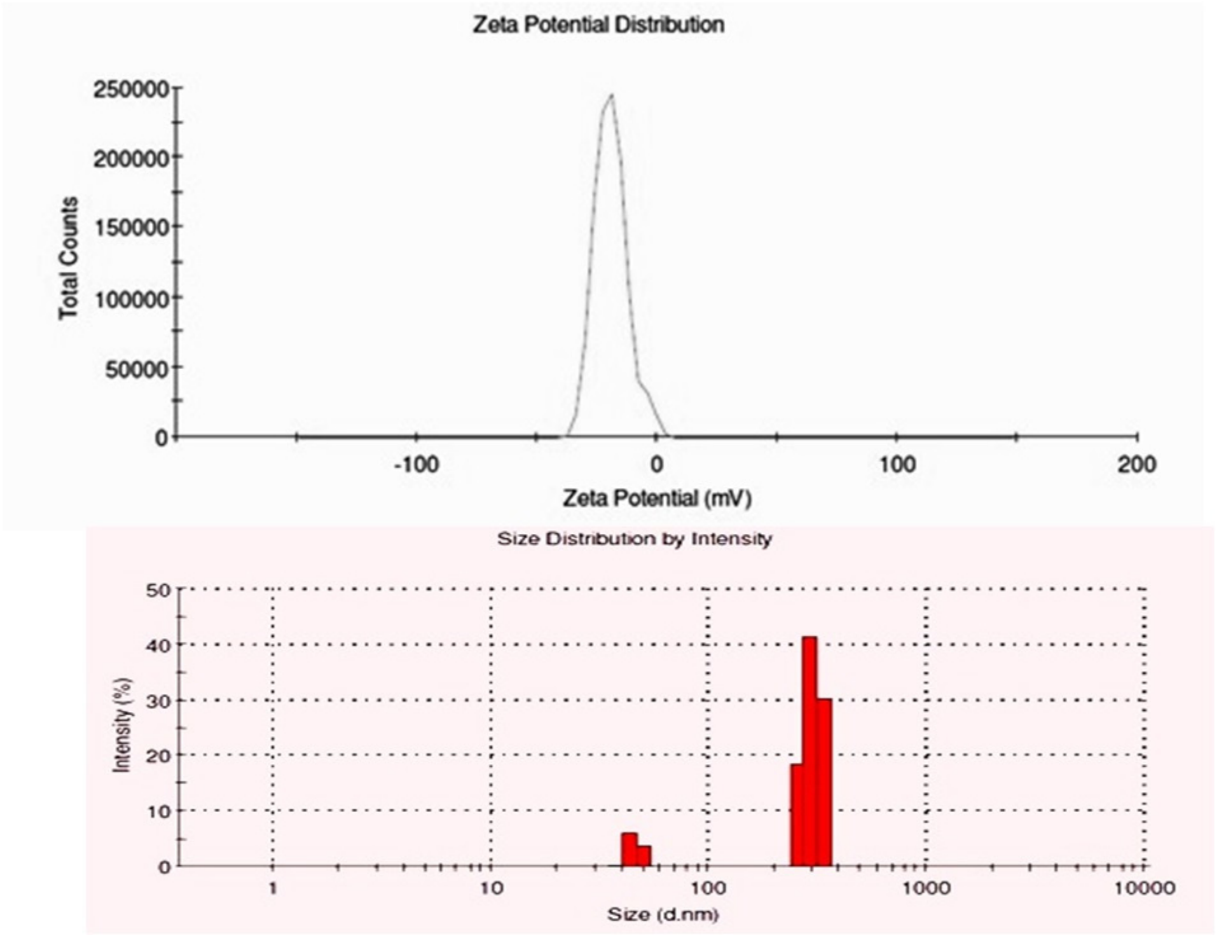

Figure S6: Zeta potential and DLS of green synthesized copper nanoparticles

Table S1: FT-IR functional group's identification of green cluster copper nano-pesticide synthesized from entomopathogenic fungi *M. robertsii*

S.no	Vibrational assignment	Observed wavenumber (cm^{-1})	Functional group	Visible intensity
1	ArO-H H bonded	3441.90	Phenols	Broad peak
2	-CH2-	2831.11	Alkanes	Medium small sharp peak
3	Ar-CH=CHR	2400.61	Alkenes	Medium peak
4	C-O stretching	1600.18	Carboxylic acids	Small broad peak
5	C-H out of plane	1000.61	Aromatics	Medium peak
6	=CH out of plane	820.15	Alkenes	Medium peak
7	S-S disulfide asym	500.18	Misc	Medium sharp peak
